# The Nature and Treatment of Dropsy

**Published:** 1908-02

**Authors:** Thomas Hunt Stucky

**Affiliations:** Louisville, Ky., Formerly Professor of Theory and Practice of Medicine and Clinical Medicine, Hospital College of Medicine; Ex-President Mississippi Valley Medical Association


### The Nature and Treatment of Dropsy.

BY DR. THOMAS HUNT STUCKY, A. M., M. D., LOUISVILLE, KY.,
Formerly Professor of Theory and Practice of Medicine and Clinical Medicine, Hospital College of Medicine; Ex-President Mississippi Valley Medical Association.

The term dropsy is one which is rather loosely applied to any abnormal accumulation of watery fluid in the body. In more concise technical language we use the word edema when the fluid occurs within the interstices of tissue and ascites when it is free in the abdominal cavity. For accumulations in other cavities special names are used, as hydrothorax, hydropericardium, hydrocephalus, etc.

Edema of the subcutaneous cellular tissue is a very common form of dropsy; it is usually confined to certain regions, but may become general, in which case it is referred to as anasarca. The site in which edema most frequently occurs is in the feet and ankles; next to this is the region about the lower eyelids.

In a normal state of health there constantly exudes from the blood vessels, especially the capillaries, a fluid derived from the serum of the blood. This percolates between the cells of all tissues, and is the means of carrying nourishment to all those cells which are not in direct contact with blood vessels. These interstices between the cells are drained by the vessels of the lymphatic system, which are able to carry off easily all the fluid which comes to them under ordinary circumstances.

The composition of this lymph is very nearly the same as that of the serum of the blood. It contains a larger proportion of water and only about 3 per cent of proteids. As a rule, it contains no fibrin, although it will coagulate upon the addition of fibrin ferment, showing the presence of some of the fibrin elements. Its specific gravity will average about 1.015.

Cohnheim taught that the most important factor requisite to the production of dropsy is malnutrition of the walls of the blood vessels. It is well to keep this in mind, for although in most cases the other causative agents are much more evident yet this element of malnutrition will almost certainly be found to exist in a greater or less degree. It can readily be imagined that the single layer of delicate cells which form the wall of the capillary vessels would be very sensitive to changes in the blood which is constantly passing them. In diseases of the kidneys, the liver and other excretory organs a number of poisonous matters accumulate in the blood to an abnormal degree, and they can not fail to harm this lining membrane. Fevers of all kinds, the presence of bacteria, pus or other products of microbic invasion will produce the same effect. Of course, in these diseases we do not often have dropsy, but the endothelial membrane is in a weakened condition, and a comparatively slight determining cause will bring on this symptom.

An excess of blood in the part may produce an edema. When it is caused by overdistension of the arteries the liability to edema is not so great as when there is a venous stasis. In the former case the capillaries are constantly receiving plenty of fresh blood, while in the latter the same blood remains in the vessels and the endothelium derives but little benefit from it. Valvular disease of the heart is one of the main causes of a general slowing of the blood current and especially of the venous stasis. The pressure from behind, the vis a tergo, of the blood in the veins is never great under the best circumstances, and when the power of the heart is weakened from any cause this propelling force is reduced to a very low point. In valvular disease of the heart the amount of blood passed through this organ is diminished until a compensatory hypertrophy is established. In the meantime the blood in the veins being impeded to some extent in front and not forced from behind, flows very sluggishly.

In the legs the blood has to mount some distance against the force of gravity, which greatly retards the circulation. While the patient walks about vigorously the contraction of the muscles of the legs, by pressing upon the vessels, helps to force the blood forward; but such patients can rarely walk with any great amount of vigor, the blood does not receive this extra help, and an edema of the feet and ankles is almost sure to be seen at some stage of the case. At first this is merely a puffiness observed at night after being up all day. The condition spreads from the feet up the legs, becoming greater as the case progresses. After a time the swelling is not entirely reduced even after a night's rest; the parts feel cold and pit upon pressure, the dents remaining for quite a length of time. Unless the case is treated properly this edema will finally involve a large portion of the body, it is an expression of extreme weakness of the circulation, and is rightly regarded by the laity as a very grave symptom.

Allied to this condition in certain ways is the accumulation of fluid in the abdomen. This escapes from the vessels of the mesentery and intestines; it is seen in conjunction with dropsy in other parts, and may come from the same state of cardiac weakness which causes edema of the feet. In many instances, however, it has a special cause in an obstruction to the flow of blood through the liver. As is well known, all of the blood from the stomach, small intestine and most of the colon is gathered up into the portal vein and goes through the liver. Whenever there is any obstruction in this organ the blood is dammed back into the other viscera and its fluid oozes out into the general peritoneal cavity.

A state of inflammation of the liver, bringing an abundance of leucocytes to the organ and causing a swelling of its cellular portion, produces a compression of the blood vessels, thus impeding the circulation. In chronic inflammations the overgrowth of the connective tissue has the same effect but it is carried to a greater degree. In this disease the lumen of the blood vessels is sometimes almost obliterated, and it is in such cases that we find persistent dropsy in the abdomen.

In diseases of the kidneys a large proportion of the poisonous matters which these organs should excrete are allowed to remain in the blood. These injure the walls of the capillaries, as referred to above, and the dropsy first seen in these cases is probably purely from this cause. Later on these same poisons affect the force of the heart muscle and weaken the nervous system, so that we may readily see how a part of the general anasarca sometimes observed in such cases may be due to circulatory weakness.

The general treatment of these cases depends, of course, largely upon the cause. Where the heart alone is at fault the patient should be put to bed, or at least confined to a chair if he can not lie down with comfort, as frequently happens. Efforts must be made to increase the nutrition of the heart muscle and the efficiency of its nerve supply. To this end we must attend to his diet; such patients have a poor digestion and are often afflicted with diarrhea. The most nourishing and easily digested foods must be given, in as great an amount and at as short intervals as he can digest them. Tonics for the heart and nerve must be administered, but care and judgment must be observed in their use, for there is grave danger of stimulating the heart beyond its strength.

Digitalis and strychnine are powerful drugs, and their effects must be watched constantly. I have had better results with a preparation called anasarcin, which is a combination of several mild tonics, alteratives and diuretics. It is a much safer drug to leave with the patient, and is very reliable in its action; its diuretic effect is one of the best means of reducing the dropsy.

Cases of renal dropsy are frequently helped by the saline purgatives and by any measures which increase the amount of perspiration. The poisons which should be eliminated by the kidney can in a large measure escape through the bowel and the skin, and these means must be used to the point of endurance. As the circulation is always weak it is necessary to administer cardiac and general tonics, and for these patients the same drug, anasarcin, is probably more efficacious than any other.

For dropsy into the abdomen the diuretic action of anasarcin and its tonic effect make it the remedy on which I have depended.

This seems high praise, but the favorable opinion formed will be found in the histories of cases given below, which are only a few chosen at random from my practice during the past year:

Case 1.—Mrs. E., married; physical examination, aortic stenosis; no doubt of long standing. Goitre from pubescence. During first pregnancy developed acute nephritis with pronounced general anasarca. Usual remedies tried unsuccessfully. Anasarcin recommended; tabled to be used every three hours. After three or four days' treatment, marked diminution of the dropsical effusion, which, being carried on during the remainder of gestation, enabled her to go through the parturient period successfully. There have been several returns of the dropsy since this attack three years ago, all of which have yielded promptly by the use of this remedy.

CASE 2.—Mrs. F., widow, age 67 years. Mitral regurgitation. Chronic interstitial nephritis; general anasarca, with all accompanying and distressing symptoms. Diuretin, infusion digitalis and the general agents proved unsuccessful. Anasarcin, one tablet every three hours encouraged by the use of salines produced decided relief to such an extent that the patient is now going about in comparative comfort.

CASE 3.—Edwin R., aged 18 years. History of scarlet fever when 7 years of age. Aortic regurgitation during attack of scarlet fever with general anasarca, which only partially disappeared after months of confinement with the usual remedies. Was placed upon anasarcin six weeks ago; one tablet every three hours until active elimination was cured both by kidney and bowels; then a tablet three times a day. At this time, August 2, 1907, there is no evidence of any dropsy, and to all appearances he seems to be regaining his strength rapidly.

CASE 4.—John R., 42 years of age. Has been a spreer. Hypertrophic cirrhosis of the liver. Mitral regurgitation; albumen, hyaline casts and granular casts found in the urine. The effusion was so great into the abdominal cavity that it was necessary to aspirate. Four gallons of fluid withdrawn, producing very decided relief, showing evidence a few days thereafter of the return of the edema. He was placed upon anasarcin as above described, in connection with tonics, and has been comparatively comfortable ever since. My observation has been that this agent is serviceable in all dropsical conditions, irrespective of cause—with the exception probably of those which are entirely mechanical in character.—*Medical Progress, Louisville, Ky.*

